# Supplementary material for: Predicting cognitive function 3 months after surgery in patients with a glioma
Source: Neurooncol Adv. 2025 May 2;7(1):vdaf081. doi: 10.1093/noajnl/vdaf081 (PMC12201988; doi:10.1093/noajnl/vdaf081)
Supplement: vdaf081_suppl_Supplementary_Materials [file vdaf081_suppl_supplementary_materials.zip › Online Supplement - Reporting checklist.docx]

## Key reporting points Bayesian Analysis Reporting Guidelines

1. Explain the model
   1. Data variables ✔
   2. Likelihood function and parameters ✔
   3. Prior distribution ✔
   4. Formal specification ✔
   5. Prior predictive check ✔
2. Report details of the computation
   1. Software ✔
   2. MCMC chain convergence ✔
   3. MCMC chain resolution ✔
3. Describe the posterior distribution
   1. Posterior predictive check ✔
   2. Summarize posterior of variables ✔
   3. BF and posterior model probabilities: NA.
4. Report decisions (if any) and their criteria: N.A
5. Report sensitivity analysis
   1. For broad priors ✔
   2. For informed priors: N.A.
   3. For default priors ✔
   4. BFs and model probabilities: N.A.
   5. Decisions: N.A.
6. Make it reproducible
   1. Software and installation ✔
   2. Software version details ✔
   3. Script and data ✔
   4. Readable for humans ✔
   5. All auxiliary files ✔
   6. Runs as posted ✔
   7. MCMC chains for time-intensive runs: N.A.
   8. Reproducible MCMC ✔
